# Supplementary material for: An OpenStreetMap derived building classification dataset for the United States
Source: Sci Data. 2024 Nov 9;11:1210. doi: 10.1038/s41597-024-04046-w (PMC11550320; doi:10.1038/s41597-024-04046-w)
Supplement: Supplementary file 1 — Supplementary information [file 41597_2024_4046_MOESM1_ESM.pdf]

An OpenStreetMap derived building classification dataset for the United States (Supplementary Information)

Henrique F. de Arruda[1,*], Sandro M. Reia[1], Shiyang Ruan[1], Kuldip S. Atwal[1], Hamdi Kavak[2], Taylor Anderson[1], and Dieter Pfoser[1]

[1]Geography and Geoinformation Science, College of Science, George Mason University, 4400 University Dr., Fairfax, 22030, VA, U.S.
[2]Center for Social Complexity, College of Science, George Mason University, 4400 University Dr., Fairfax, 22030, VA, U.S.
[*]corresponding author: Henrique F. de Arruda (h.f.arruda@gmail.com)

Contents

S1 Selected auxiliary information **1**
S1.1 Download data 1
S1.2 Accomodation tags 1
S1.3 Non-residential tags 2
S1.4 Selected auxiliary information lists 2
Skipped tags • Residential buildings • non-residential buildings
S2 Defining UTM **3**
S3 Ground truth **4**
S3.1 Landuse information to residential or non-residential 4
S4 No sheds and garages **11**
S5 Proportion of buildings without annotations **11**
S6 Select particular footprints **11**
References **11**

S1 Selected auxiliary information

In this section, we divide the building tags into residential and non-residential categories. In addition, we show the tags of the auxiliary information used for those buildings that are not classified by the building tags.

S1.1 Download data

In order to download data using OSMnx, we must specify which keys we have used. The set of keys used here is as follows. In OSMnx, one of the parameters of the method used to download data (*features_from_polygon*) is called *tag*, which specifies the OSM keys. This parameter allows the method to download all data elements that contain tags with the specified keys. The key *surface* is only used to reduce errors caused by trying to download empty data and is removed for other analyses.

- building;
- surface;
- amenity;
- emergency;
- healthcare;
- landuse;
- military;
- office;
- public_transport;
- service;
- shop;
- sport;
- telecom;
- tourism;
- brand;
- clothes;
- leisure;
- cemetery.

S1.2 Accomodation tags

Here, we list the building values for the *building* key, indicating that the tag is residential.

- apartments;
- barracks;
- bungalow;
- cabin;
- detached;
- dormitory;
- farm;
- ger;
- house;
- houseboat;
- residential;
- semidetached_house;
- static_caravan;
- stilt_house;
- terrace;
- tree_house;
- trullo;
- townhouse;
- townhome;
- boathouse;
- shed;
- garage;
- garages.

S1.3 Non-residential tags

The set of non-residential tags consists of all other building tag values not in Section S1.2, except "yes". The "yes" value represents that the building is of an unknown type.

S1.4 Selected auxiliary information lists

This set is divided into two sublists, one for residential and one for non-residential buildings.

S1.4.1 Skipped tags

Our method ignores the following tags. For the key *landuse*, if the value is "forest" the tag is skipped, and for the key "leisure", if the value is "park" or "swimming_pool" the tag is skipped. The following set of values are skipped for all keys:

- construction;
- driveway;
- grass;
- farmyard;
- farmland;
- nature_reserve.

S1.4.2 Residential buildings

For the key *landuse*, we only consider the value *residential*. Next, for the key *tourism*, we consider *apartment* and *guest_house*. Note that our method considers the key *landuse* first.

S1.4.3 non-residential buildings

The non-residential keys and their values are listed below. The values are considered in the order in which they appear in the list.

- **landuse**:
  - commercial;
  - retail;
  - industrial;
  - institutional;
  - education;
  - military;
  - port;
  - religious;
  - winter_sports;
  - cemetery;
  - grave_yard.
- **amenity**:
  - courthouse;
  - fire_station;
  - police;
  - post_depot;
  - post_office;
  - prison;
  - ranger_station;
  - townhall;
  - college;
  - kindergarten;
  - library;
  - research_institute;
  - school;
  - university;
  - car_rental;
  - car_wash;
  - vehicle_inspection;
  - ferry_terminal;
  - fuel;
  - hospital;
  - brothel;
  - casino;
  - cinema;
  - conference_centre;
  - events_venue;
  - exhibition_centre;
  - love_hotel;
  - nightclub;
  - planetarium;
  - theatre;
  - bar;
  - restaurant.

The buildings that are not classified with the previous tags and that contain a non-null value with the following keys are considered non-residential.

- emergency;
- healthcare;
- landuse;
- military;
- office;
- public_transport;
- service;
- shopv;
- sport;
- telecom;
- tourism;
- brand;
- clothes;
- leisure;
- cemetery.

S2 Defining UTM

All the data generated from this study is projected using the Universal Transverse Mercator (UTM) Coordinate Reference System (CRS). To find the best coordinates to project, we used the Python function shown in Figure S1, in which the input is a *GeoDataFrame* of *Geopandas*. For more information, see Section 6.3 of ref.[1].

```
def get_utm_crs_from_geodataframe(gdf):
    """
    Determine the appropriate UTM CRS for a given GeoDataFrame.

    Parameters:
        gdf: GeoDataFrame with the input geometries

    Returns:
        utm_crs: The EPSG code for the appropriate UTM zone
    """
    centroid = gdf.unary_union.centroid
    lon, lat = centroid.x, centroid.y
    # Get the UTM zone
    utm_zone = int((lon + 180) // 6) + 1

    # Construct the EPSG code
    if lat >= 0: #north hemisphere
        epsg_code = 32600 + utm_zone
    else: #south hemisphere
        epsg_code = 32700 + utm_zone

    return epsg_code
```

Figure S1. Python Function to calculate the UTM coordinate from the Geopandas *GeoDataFrame*.

S3 Ground truth

S3.1 Landuse information to residential or non-residential

For each dataset, we list the original land use information and its respective classification as residential (“RES”), non-residential (“NON_RES”), or mixed-use (“N/A”). Note that for those that are set to a primary use but can allow mixed-use, we set them as “N/A”.

- **Minneapolis and St. Paul:**
  - Agricultural: N/A;
  - Airport or Airstrip: NON_RES;
  - Extractive: N/A;
  - Farmstead: N/A;
  - Golf Course: NON_RES;
  - Industrial or Utility: NON_RES;
  - Institutional: NON_RES;
  - Major Highway: N/A;
  - Major Railway: N/A;
  - Manufactured Housing Park: RES;
  - Mixed Use Commercial: NON_RES;
  - Mixed Use Industrial: NON_RES;
  - Mixed Use Residential: N/A;
  - Multifamily: RES;
  - Office: NON_RES;
  - Open Water: N/A;
  - Park, Recreational, or Preserve: N/A;
  - Retail and Other Commercial: NON_RES,
  - Seasonal/Vacation: N/A;
  - Single Family Attached: RES;
  - Single Family Detached: RES;
  - Undeveloped: N/A.

  The column used to extract the land use tag is “DESC2020”.

- **Baltimore, MD:**
  - AGRICULTURAL VACANT: N/A;
  - AGRICULTURE: N/A;
  - AIRPORT: NON_RES;
  - ASSISTED LIVING FACILITY: N/A;
  - CEMETARY W/O PLACE OF WORSHIP: N/A;
  - COLLEGE: NON_RES;
  - COMMERCIAL: NON_RES;
  - COUNTY OPEN SPACE: N/A;
  - COUNTY PARK: N/A;
  - COUNTY SENIOR CENTER: N/A;
  - ELECTRIC, GAS, TELECOMMUNICATIONS UTILITY: N/A;
  - FIRE FACILITY: NON_RES;

- FURTHER REVIEW: N/A;
- HOA/COA/DEVELOPER/MULTIFAMILY MGMT: RES;
- HOSPITAL: NON_RES;
- INDUSTRIAL: NON_RES;
- LANDFILL: N/A;
- LIBRARY: NON_RES;
- MISC. GOVERNMENT-PUBLIC: NON_RES;
- MISC. INSTITUTION-PRIVATE: NON_RES;
- MIXED OFFICE/INDUSTRIAL: NON_RES;
- MIXED OFFICE/INDUSTRIAL/RETAIL: NON_RES,
- MIXED OFFICE/RETAIL: NON_RES,
- MIXED RESIDENTIAL/OFFICE/RETAIL: N/A;
- MULTI SFD: RES;
- MULTIFAMILY: RES;
- NON-COUNTY PARCEL: RES;
- OFFICE: NON_RES;
- OTHER GOVERNMENT OPEN SPACE: N/A;
- OTHER PRIVATE OPEN SPACE: N/A;
- OTHER PUBLIC PARK: N/A;
- PARK AND RIDE: N/A;
- PERMANENT EASEMENT: N/A;
- PIPELINE: N/A;
- PLACE OF WORSHIP: N/A;
- POLICE FACILITY: NON_RES;
- PRIVATE SCHOOL: NON_RES;
- PRIVATELY OWNED GOLF COURSE: NON_RES;
- PUBLIC SCHOOL OR SCHOOL SITE: NON_RES;
- PUBLICLY OWNED GOLF COURSE: NON_RES;
- RAIL: N/A;
- RESERVOIR PROPERTY: N/A;
- ROAD: N/A;
- RURAL RESIDENTIAL SFD: N/A;
- SFA: RES;
- SFD: RES;
- SFSD: RES;
- STATE PARK: N/A;
- STORM DRAINAGE: N/A;
- UNBUILDABLE/ENVIRONMENTALLY CONSTRAINED: N/A;
- VACANT: N/A;
- WATER: N/A;
- WATER OR SEWER UTILITY: N/A.

The column used to extract the land use tag is: “GIS_LU_COD”.

- **Boulder, CO:**
  - Agricultural: N/A;
  - Commercial: NON_RES;
  - Foundation/Ruin: N/A;
  - Garage/Shed: N/A;
  - Industrial: NON_RES;
  - Medical: NON_RES;
  - Misc: N/A;
  - Parking Structure: N/A;
  - Public: NON_RES;
  - Public Safety: NON_RES;
  - Religious: NON_RES;
  - Residential: RES,
  - School: NON_RES;
  - Tank: N/A.

  The column used to extract the land use tag is “BLDGTYPE”.
- **Fairfax, VA:**
  - High-density Residential: RES;
  - Low-density Residential: RES;
  - Medium-density Residential: RES;
  - Agricultural: N/A;
  - Commercial: NON_RES;
  - Industrial, light and heavy: NON_RES;
  - Institutional: NON_RES;
  - Open land, not forested or developed: N/A;
  - Public: NON_RES;
  - Recreation: NON_RES;
  - Surface water: N/A;
  - Utilities: N/A;
  - Industrial: NON_RES;
  - Institutional - General: NON_RES;
  - Institutional - Government: NON_RES;
  - Mixed-Use Residential/Commercial: N/A;
  - Open Space - Private: N/A;
  - Open Space - Public: N/A;
  - Residential - Multifamily: RES;
  - Residential - Single Attached: RES;
  - Residential - Single Detached: RES;
  - Vacant: N/A;
  - None: N/A (None type).

  The column used to extract the land use tag is “CATEG” in the case of the county and “ELU” in the case of Fairfax City.

- **Hanover, VA:**
  - A-1: N/A;
  - AR-1: N/A;
  - AR-2: N/A;
  - AR-6: N/A;
  - B-1: NON_RES;
  - B-2: NON_RES;
  - B-3: NON_RES;
  - B-4: NON_RES;
  - B-O: NON_RES;
  - HE: N/A;
  - M-1: NON_RES;
  - M-2: NON_RES;
  - M-3: NON_RES;
  - MX: N/A;
  - O-S: NON_RES;
  - PMH: RES;
  - PSC: NON_RES;
  - PUD: N/A;
  - R-1: RES;
  - R-2: RES;
  - R-3: RES;
  - R-4: RES;
  - R-5: RES;
  - R-6: RES;
  - RC: N/A;
  - RM: RES;
  - RO-1: N/A;
  - RR-1: N/A;
  - RRC: N/A;
  - RS: RES;
  - See Map: N/A;
  - None: N/A.

  The column used to extract the land use tag is: "ZONING_LIS".

- **Mecklenburg, NC:**
  - 100 YEAR FLOOD PLAIN - AC: N/A;
  - 100 YEAR FLOOD PLAIN - LT: N/A;
  - AGRICULTURAL - COMMERCIAL PRODUCTION: N/A;
  - AIR RIGHTS PARCEL: N/A;
  - AIRPORT: NON_RES;
  - AUTO SALES AND SERVICE: NON_RES;

- BANK: NON_RES;
- BILL BOARD: N/A;
- BUFFER STRIP: N/A;
- CAR WASH: NON_RES;
- CELL TOWER: NON_RES;
- CHURCH: NON_RES;
- CLUB, LODGES, UNION HALL, SWIM CLUB: NON_RES;
- COLLEGE - PUBLIC: NON_RES;
- COMMERCIAL: NON_RES;
- COMMERCIAL COMMON AREA: NON_RES;
- COMMERCIAL CONDOMINIUM: NON_RES;
- COMMERCIAL CONDOMINIUM COMMON AREA: NON_RES;
- COMMERCIAL SERVICE(LAUNDRY,TV,RADIO,ETC): NON_RES;
- COMMERCIAL WATER FRONTAGE: NON_RES;
- CONDO AFFORDABLE HOUSING: RES;
- CONDOMINIUM: RES;
- CONDOMINIUM COMMON AREA: RES;
- CONDOMINIUM HIGH RISE: RES;
- CONDOMINIUM WATER FRONTAGE: RES;
- CONDOMINIUM WATER VIEW: RES;
- CONSERVATION - AGRICULTURAL COMM: N/A;
- CONSERVATION - FORESTRY COMM: N/A;
- CONSERVATION - WILDLIFE: N/A;
- CONSERVATION - WOODLAND EXCESS AC: N/A;
- CONVENIENCE STORE: NON_RES;
- CONVIENCE/FAST FOOD STORE: NON_RES;
- COUNTRY CLUB: NON_RES;
- DAY CARE CENTER: NON_RES;
- DEPARTMENT STORE: NON_RES;
- ENVIRONMENTAL HAZARD: NON_RES;
- FAST FOOD: NON_RES;
- FIRE DEPARTMENT: NON_RES;
- FLUM/SWIM FLOODWAY (NO BUILD ZONE): N/A;
- FOREST - COMMERCIAL PRODUCTION: N/A;
- FUNERAL (MORTUARY, CEMETERY, CREMATORIUM, MAUS): NON_RES;
- GOLF COURSE CLASS 1 - CHAMPIONSHIP: NON_RES;
- GOLF COURSE CLASS 2 - PRIVATE CLUB: NON_RES;
- GOLF COURSE CLASS 3 - SEMI-PRIVATE & MUNICIPAL: NON_RES;
- GOLF COURSE CLASS 4 - MINIMUM QUALITY: NON_RES;
- GREENWAY TRAIL: N/A;
- HABITAT FOR HUMANITY: N/A;
- HOME FOR THE AGED: RES;

- HORTICULTURAL - COMMERCIAL PRODUCTION: N/A;
- HOSPITAL, PRIVATE: NON_RES;
- HOSPITALS - PUBLIC: NON_RES;
- HOTEL/MOTEL < 7 FLOORS: NON_RES;
- HOTEL/MOTEL > 6 FLOORS: NON_RES;
- INDUSTRIAL: NON_RES;
- INDUSTRIAL COMMON AREA: NON_RES;
- INDUSTRIAL PARK: NON_RES;
- INSTITUTIONAL: NON_RES;
- ISLAND: N/A;
- LABORATORY / RESEARCH: NON_RES;
- LEASEHOLD INTEREST: NON_RES;
- LIGHT MANUFACTURING: NON_RES;
- LUMBER YARD: NON_RES;
- MARINA LAND: NON_RES;
- MEDICAL CONDOMINIUM: NON_RES;
- MEDICAL CONDOMINIUM COMMON AREA: NON_RES;
- MEDICAL OFFICE: NON_RES;
- MINI WAREHOUSE: NON_RES;
- MINIATURE GOLF COURSES/DRIVING RANGE: NON_RES;
- MINING: N/A;
- MOBILE HOME HS: RES;
- MOBILE HOME PARK: N/A;
- MOBILE HOME SUBDIVISION: N/A;
- MULTI FAMILTY AFFORDABLE HOUSING: RES;
- MULTI FAMILY: RES;
- MULTI FAMILY COMMON AREA: RES;
- MULTI FAMILY DUPLEX/TRIPLEX: RES;
- MULTI FAMILY GARDEN: N/A;
- MULTI FAMILY HIGH RISE: RES;
- MULTI FAMILY TOWNHOUSE: RES;
- MULTI FAMILY WATER ACCESS: N/A;
- MUNICIPAL AIRPORT: NON_RES;
- MUNICIPAL EDUCATION: NON_RES;
- NEW PARCEL: N/A;
- NO LAND INTEREST: N/A;
- NURSING HOME: RES;
- OFFICE: NON_RES;
- OFFICE CONDOMINIUM: NON_RES;
- OFFICE CONDOMINIUM COMMON AREA: NON_RES;
- OFFICE HIGH RISE - > 6 STORIES: NON_RES;
- OTHER COUNTY PROPERTY: NON_RES;

- OTHER FEDERAL: NON_RES;
- OTHER MUNICIPAL: NON_RES;
- PACKING PLANT: NON_RES;
- PARKING: N/A;
- PATIO HOME: N/A;
- PATIO HOME - WATERFRONT: N/A;
- PETROLEUM AND GAS: N/A;
- PVT Owned RR with Rail ROW: N/A;
- R101: N/A;
- REC AREA: N/A;
- RESERVED PARCEL: N/A;
- RESIDENTIAL AFFORDABLE HOUSING: RES;
- RESTAURANT: NON_RES;
- RIGHT OF WAY: N/A;
- ROADWAY CORRIDOR: N/A;
- RURAL HOMESITE: N/A;
- SCHOOL - PUBLIC: NON_RES;
- SCHOOL,COLLEGE, PRIVATE: NON_RES;
- SERVICE GARAGE: NON_RES;
- SERVICE STATION: NON_RES;
- SHOPPING CENTER - MALL: NON_RES;
- SHOPPING CENTER - STRIP: NON_RES;
- SINGLE FAMILY RESIDENTIAL: RES;
- SINGLE FAMILY RESIDENTIAL - ACREAGE: RES;
- SINGLE FAMILY RESIDENTIAL - COMMON: RES;
- SINGLE FAMILY RESIDENTIAL - GOLF: RES;
- SINGLE FAMILY RESIDENTIAL - RIVER: RES;
- SINGLE FAMILY RESIDENTIAL - WATER VIEW: RES;
- SINGLE FAMILY RESIDENTIAL - WATERFRONT: RES;
- SINGLE FAMILY RESIDENTIAL GATED COMMUNITY: RES;
- SINGLE FAMILY RESIDENTIAL MINI FARM/ESTATE: RES;
- STATE PROP: N/A;
- SUBMERGED LAND, RIVERS AND LAKES: N/A;
- SUPERMARKET: NON_RES;
- TOWN HOUSE GOLF COURSE FRONTAGE: RES;
- TOWN HOUSE SFR: RES;
- TOWN HOUSE WATER ACCESS: RES;
- TOWN HOUSE WATER FRONTAGE: RES;
- TOWN HOUSE COMMON AREA: RES;
- TOWNHOUSE AFFORDABLE HOUSING: RES;
- TRUCK TERMINAL: NON_RES;
- UNSUITABLE FOR SEPTIC: N/A;

- USE VALUE HOMESITE: N/A;
- UTILITY (GAS, ELECTRIC, TELEPHONE, TELEGRAPH, RAIL: N/A;
- UTILITY EASEMENT: N/A;
- UTILITY/P: N/A;
- WAREHOUSE CONDOMINIUM: NON_RES;
- WAREHOUSE CONDOMINIUM COMMON AREA: NON_RES;
- WAREHOUSING: NON_RES;
- WASTELAND, SLIVERS, GULLIES, ROCK OUTCROP: N/A,
- WATER PLANT: N/A;
- WATER RETENTION POND: N/A;
- WELL LOT: N/A;
- WETLAND: N/A;
- WOODLAND - EXCESS ON AG PCL: N/A.

The column used to extract the landuse tag is: "landuse_de".

S4 No sheds and garages

Here are the results of the comparison to the ground truth, where we exclude the sheds and garages. Table S1 refers to Minneapolis and St. Paul, and Table S2 refers to the other regions of the U.S.

County	Class	Precision	Recall	F1-Score	Avg. F1-Score
Anoka, MN	non-residential	0.99	0.72	0.83	0.89
	residential	0.90	1.00	0.95	
Carver, MN	non-residential	0.96	0.75	0.84	0.91
	residential	0.96	1.00	0.98	
Dakota, MN	non-residential	0.98	0.77	0.86	0.93
	residential	0.98	1.00	0.99	
Hennepin, MN	non-residential	0.97	0.77	0.86	0.92
	residential	0.97	1.00	0.98	
Ramsey, MN	non-residential	0.97	0.63	0.76	0.86
	residential	0.94	1.00	0.97	
Scott, MN	non-residential	0.98	0.70	0.81	0.89
	residential	0.95	1.00	0.97	
Washington, MN	non-residential	0.98	0.76	0.86	0.92
	residential	0.96	1.00	0.98	

Table S1. Prediction results for the Minneapolis and St. Paul area excluding sheds and garages.

S5 Proportion of buildings without annotations

Figure S2 shows the proportion of untagged buildings per county in the contiguous U.S.

S6 Select particular footprints

Figure S3 shows the Python code to select only the footprints that are not sheds, garages, and parking lots.

References

1. Lovelace, R., Nowosad, J. & Muenchow, J. *Geocomputation with R* (Chapman and Hall/CRC, 2019).

Region	Class	Precision	Recall	F1-Score	Avg. F1-Score
Baltimore, MD	non-residential	0.94	0.83	0.88	0.94
	residential	0.99	1.00	0.99	
Boulder, CO*	non-residential	0.85	0.70	0.77	0.88
	residential	0.97	0.99	0.98	
Fairfax, VA**	non-residential	0.96	0.83	0.89	0.94
	residential	0.99	1.00	0.99	
Hanover, VA	non-residential	0.97	0.62	0.75	0.87
	residential	0.97	1.00	0.98	
Mecklenburg, NC	non-residential	0.92	0.74	0.82	0.91
	residential	0.98	1.00	0.99	

Table S2. Prediction results for other regions of the U.S. excluding sheds and garages. Here, Boulder* is a city, Fairfax** is both the county and the town of Fairfax, and the others are counties.

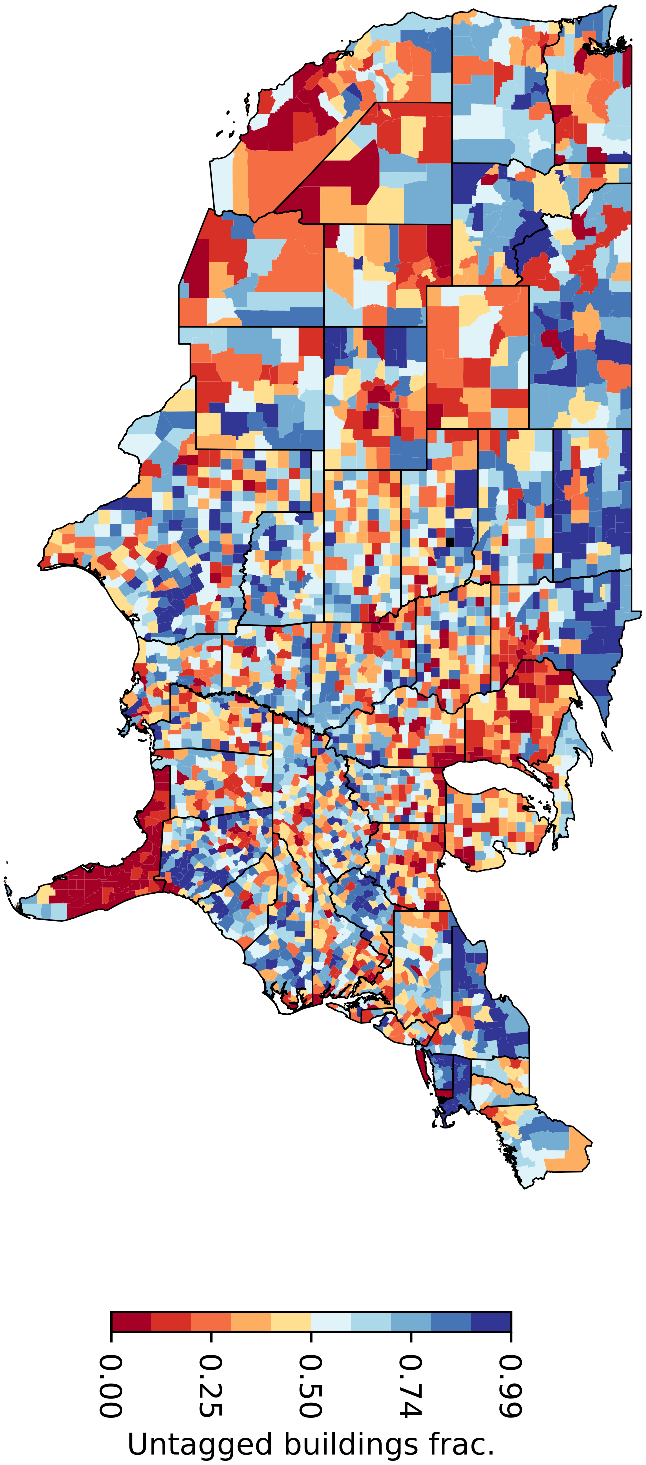

Figure S2. Map of the contiguous U.S. with the proportion of untagged buildings per county or equivalent region. The color black indicates a county for which data are not available in OSM (Wheeler County, NE).

```
...
delete = ["building:shed", "building:garage", "building:garages", "building:parking"]
buildings_df = buildings_df[~buildings_df['tag used'].isin(delete)]
...
```

Figure S3. Code to select only the footprints that are not sheds, garages, and parking lots. Python code to remove the rows of sheds, garages, and parking lots from the Geopandas *GeoDataFrame*.
